# Animal Models for Autoimmune Hepatitis: Are Current Models Good Enough?

**DOI:** 10.3389/fimmu.2022.898615

**Published:** 2022-07-12

**Authors:** Urs Christen, Edith Hintermann

**Affiliations:** Pharmazentrum Frankfurt/Zentrum für Arzneimittelforschung, Entwicklung und Sicherheit (ZAFES), Goethe University Hospital, Frankfurt am Main, Germany

**Keywords:** CYP2D6 model, humanized mice, wildling mice, natural environment, translation, clinical trial

## Abstract

Autoimmune liver diseases like autoimmune hepatitis, primary biliary cholangitis, primary sclerosing cholangitis, and IgG4-related cholangitis are chronic inflammatory diseases of the liver with an autoimmune background. The therapy of autoimmune hepatitis targets the autoreactive immune system and is largely dependent on the use of glucocorticoids and cytostatic drugs. In contrast, the treatment of cholestatic autoimmune liver diseases is restricted to the use of secondary or semi-synthetic bile acids, like ursodeoxycholic acid or obeticholic acid. Although the management of the disease using such drugs works well for the majority of patients, many individuals do not respond to standard therapy. In addition, chronic treatment with glucocorticoids results in well-known side effects. Further, the use of bile acids is a symptomatic therapy that has no direct immunomodulatory effect. Thus, there is still a lot of room for improvement. The use of animal models has facilitated to elucidate the pathogenesis of autoimmune liver diseases and many potential target structures for immunomodulatory therapies have been identified. In this review, we will focus on autoimmune hepatitis for which the first animal models have been established five decades ago, but still a precise treatment for autoimmune hepatitis, as obtainable for other autoimmune diseases such as rheumatoid arthritis or multiple sclerosis has yet to be introduced. Thus, the question arises if our animal models are too far from the patient reality and thus findings from the models cannot be reliably translated to the patient. Several factors might be involved in this discrepancy. There is first and foremost the genetic background and the inbred status of the animals that is different from human patients. Here the use of humanized animals, such as transgenic mice, might reduce some of the differences. However, there are other factors, such as housing conditions, nutrition, and the microbiome that might also play an important role. This review will predominantly focus on the current status of animal models for autoimmune hepatitis and the possible ways to overcome discrepancies between model and patient.

## Autoimmune Liver Diseases

Current therapies of autoimmune liver diseases (ALD) are either dependent on the use of glucocorticoids and cytostatic drugs to dampen the autoreactive immune system or are restricted to the use of secondary or semi-synthetic bile acids, like ursodeoxycholic acid or obeticholic acid to treat cholestatic autoimmune liver diseases. In the last two to three decades many review articles focusing on animal models for ALD from us and others listed the problems that have to be overcome to break immune tolerance in the liver, reported the method of action of available models, and pointed out which factors might drive the immunopathogenic process. Such reviews then often continued a section stating that one reason for the apparent deficit in novel therapeutic interventions might be the lack of appropriate models reflecting all the aspects of human ALD. Many years have gone by since, many new models have been generated, new mechanisms of liver damage have been found, and many critical target molecules have been identified. Nevertheless, in the clinic it seems that we have been treading water as still very unspecific treatments like glucocorticoids and cytostatic drugs are utilized for immunosuppression.

ALD come in four flavors that are often diagnosed as separate entities, but sometimes comprise polyautoimmunities that manifest as complex overlap syndromes of multiple autoimmune diseases. Autoimmune hepatitis (AIH), primary biliary cholangitis (PBC), primary sclerosing cholangitis (PSC), and immunoglobulin G4 (IgG4)-associated cholangitis (IAC) are chronic inflammatory diseases of the liver with a strong autoimmune component. Whereas AIH predominantly affects the liver parenchyma, the three cholestatic diseases are characterized by a destruction of the small and/or large bile ducts. AIH is characterized by an interface hepatitis with peacemeal necrosis, hypergammaglobulemia and depending on the subtype the generation of anti-nuclear antibodies (ANA) and/or anti-smooth muscle (SMA) autoantibodies (AIH type 1) or liver-kidney microsomal antibodies type 1 (LKM-1) (AIH type 2) (1–4). PBC is characterized by an autoimmune destruction of the small intrahepatic bile ducts. Patients with PBC generated anti-mitochondrial antibodies (AMA) that predominantly react to enzymes of the 2-oxoacid-dehydrogenase family, such as the E2-subunit of the pyruvate dehydrogenase (PDH) (5). Another cholangiopathic disease is PSC that affects large intra- and extrahepatic bile ducts and is characterized by the appearance of concentric rings of fibrotic tissue (onion-ring fibrosis) around the bile ducts (6, 7). Further, IAC as a manifestation of IgG4-related disease (IgG4-RD) is rather difficult to diagnose, but somewhat mimics sclerosing cholangitis with the presentation of mass-forming lesions and/or strictures in the biliary tract. The tissue is often infiltrated by IgG4-expressing plasma cells (8, 9).

All these forms of ALD have an unknown etiology and share the chicken-and egg dilemma with many other autoimmune diseases. It is often not clear if tissue damage is preceding autoimmunity or vice versa. Many inducible animal models target one of the two conditions, either by an attempt to break immune tolerance resulting in chronic inflammation and autoimmunity or by directly damaging liver tissue through hepatotoxins and thereby eliciting local inflammatory processes. Spontaneous models predominantly originate from transgenic mice that have inherent defects in immune regulation or display spontaneous tissue damage.

In the present review we will focus on AIH in particular, since there is a high discrepancy between the diversity of animal models for AIH that have been generated to date and the translation of the acquired knowledge into clinical practice.

## Traditional Models

In general animal models of human diseases have two major purposes. First, the elucidation of pathogenic disease mechanisms and the identification of critical factors driving the disease. Second, the evaluation of a therapeutic intervention targeting such critical factors with a possibility to translate the obtained results to a therapy of the human disease. Before we discuss the translation of the findings in animal models, we will highlight a few of these many animal models. For a more complete list and discussion of animal models for AIH, we would like to refer to two recent reviews (10, 11). In AIH the first models have been generated half a century ago. Meyer zum Büschenfelde and colleagues used human liver isolate containing two antigens, a “water soluble protein” (LP-2) and a “water insoluble macromolecular low density lipoprotein” (LP-1), to immunize rabbits that subsequently developed liver lesions characteristic for AIH (12). Those rabbits also generated antibodies to LP-1 and LP-2, which however were not pathogenic on their own as demonstrated with antibody transfer experiments (12). A similar approach of injecting liver autoantigens was used by Lohse et al. who generated an experimental autoimmune hepatitis (EAH) model (13). They injected C57BL/6 mice with a so-called S-100 fraction, which was the 100,000 g supernatant of syngeneic liver homogenate emulsified in complete Freund’s adjuvant. Interestingly, these mice developed not only a transient liver damage, characterized by perivascular cellular infiltrates and hepatocyte necrosis, but generated also S-100 protein-specific T-cells (13). Naturally, those early models for human AIH were intended to get new insight into the nature of the autoantigens and to build a basis for continuative mechanistic evaluation of the immunopathogenesis, rather than to directly test novel therapeutic interventions.

Compared to many other organs, the liver has a natural tendency for peripheral immune tolerance that seems to protect the organ from excessive autoimmunity. One has to consider here that the liver otherwise would be prone to autoimmunity due to the constant exposure to drugs and xenobiotics that might result in immune-mediated drug-induced liver injury (DILI) (14). Further, the liver encounters with many liver-tropic pathogens, such as Hepatitis-, Coxsackie- and Herpes simplex viruses, which might act as triggering factor of autoimmunity (15). Many mechanisms have been suggested of how such an immunosuppressive milieu is being established in the liver. Thereby, liver-resident cells, including hepatic stellate cells (HSC) and liver sinusoidal endothelial cells (LSEC) seem to play an important role by inducing T cell inactivation or apoptosis as well as by expanding regulatory T cells (Treg) (16–19). Lüth et al. demonstrated that the immunoregulatory function of the liver goes even beyond its own confinements. Ectopic hepatic expression of myelin basic protein (MBP), a human autoantigen in multiple sclerosis (MS), protected mice from the induction of MS-like disease in the CNS by generation of MBP-specific Treg that blocked autoaggressive T cells (20). Thus, after the initial models with crude liver homogenates, many models targeted to overcome the immunosuppressive nature of the liver by novel technologies, such as the generation of transgenic mice. The goal was to gain knowledge about the immunomodulatory event in the liver, but also to generate more precise models for AIH for the identification of critical factors driving the disease. Here, we will highlight just three examples and would like to refer again to our recent review for more detailed information (10).

One of the first transgenic models was established by Frank Chisari and colleagues who generated mice expressing the hepatitis B virus surface antigen (HBsAg) under the control of the albumin (Alb) promoter specifically in the liver. They achieved a transient hepatitis that resembled a delayed-type hypersensitivity reaction rather than AIH by an adoptive transfer of HBsAg-specific cytotoxic T-lymphocytes (CTL) that triggered apoptosis of the HBsAg expressing hepatocytes (21, 22). In a similar way Limmer et al. generated transgenic mice expressing the model antigen H-2K^b^ (a mouse MHC class I molecule) also controlled by the Alb promoter. However, instead of transferring H-2K^b^-specific T cells they generated a double transgenic mouse that also expressed H-2-K^b^-specific T cell receptors (TcR). Interestingly, such TcRxAlb.K^b^ mice only developed a transient form of AIH after an additional activation, by either an infection with the liver tropic pathogen *L. monocytogenes* or by transfer of tumor cells expressing both H-2K^b^ as well as IL-2 (23). The last example demonstrates again that the liver has a tolerogenic nature. Voehringer et al. generated transgenic mice expressing the immunodominant CD8 T cell epitope GP33 of the glycoprotein (GP) of the lymphocytic choriomeningitis virus (LCMV). As in previous models the expression of GP33 was restricted to the liver by using the Alb promoter (24). To achieve AIH-like disease the mice required not only infection by LCMV, but also the transfer of TcR transgenic GP33-specific T cells. However, even under such strong inflammatory insults the observed liver damage characterized by elevated serum aminotransferase levels and cellular infiltrates was only transient (24). This stands in contrast to the RIP-LCMV-GP model for fast-onset type 1 diabetes (T1D), in which the entire GP of LCMV is expressed under the control of the rat insulin promoter (RIP) in the β-cells of the islets of Langerhans in the pancreas (25, 26). In the RIP-LCMV-GP model an infection of mice with a low dose of LCMV (5 x 10^3^ to 1 x 10^5^ pfu) is sufficient to induce a long-lasting, chronic form of T1D (26). However, just like in the Voehringer AIH-like disease model, in the RIP-LCMV-GP model majority of autoreactive CD8 T cells are specific for the immunodominant epitope GP33. Recently, Preti et al. investigated the role of autoreactive CD4 T cells in an LCMV-related AIH model (27). They constructed a transgenic mouse in which the CLIP sequence of the CD74 has been replaced by the immunodominant CD4 epitope GP_61-80_ specifically in the liver. Thereby, the presentation of GP_61-80_ by MHC II was facilitated in the liver. These mice have been crossed with TcR-transgenic Smarta mice that predominantly carry GP_61-80_-specific CD4 T cells. Interestingly, they found that besides an activation of liver-infiltrating CD4 T cells, the mice also displayed a reduced functionality of GP-specific regulatory T cells. In combination a chronic form of AIH-like disease, with interface hepatitis, formation of autoantibodies, as well as elevated IgG and ALT levels ensued (27). Although, this model uses modified autoantigen-presentation and autoantigen-specific TcR, these data indicate that the tolerogenic environment in the liver might be overcome *via* autoreactive CD4, rather than CD8, T cells.

Knowing about the immunotolerant status of the liver, other models included the additional expression of pro-inflammatory factors, such as IFNγ, IL-2, or IL-12. In particular, IL-12 has been extensively used to boost autoimmunity. For example, Djilali-Saiah et al. induced AIH-like disease in mice expressing the nucleoprotein (NP) of LCMV as a target antigen under the control of the transthyretin (TTR) promoter in the liver by DNA-vaccination with a plasmid coding for NP. Interestingly, only the additional injection of a plasmid encoding IL-12 resulted in elevated serum aminotransferase levels and cellular infiltrations (28). In a follow-up study they used DNA vaccination with plasmids coding for the natural occurring human AIH autoantigens formiminotransferase cyclodeaminase (FTCD) and cytochrome P450 2D6 (CYP2D6). Again, they boosted autoimmunity by including sequences encoding CTLA-4 and IL-12 into the DNA-vaccination plasmid (29). Similar as in the TTR-LCMV-NP model, omitting IL-12 resulted in failure to induce AIH-like disease. Pro-inflammatory cytokines, such as IL-12 seem to play a critical role in the induction of autoimmunity in the liver. AIH-like disease, characterized by elevated serum aminotransferase levels and hypergammaglobulinemia as well as persistent cellular infiltrations, hepatic fibrosis, and even formation of ANA and anti-SMA antibodies was induced in non-transgenic wildtype C57BL/6 mice by simple injection of adeno-associated viral vector (AAV) encoding IL-12 (30). Considering the importance of IL-12 in these models it is somewhat surprising that ustekinumab, an antibody binding to the p-40 subunit present in IL-12 and IL-23, has not been used in any animal model for AIH or in AIH patients to date. Alternatively, unsuccessful studies with ustekinumab might have not been published. Anyhow, this example demonstrates where the translational problem might be located. It seems that many animal models for AIH have been developed in the past, but only very few of them have been used to evaluate actual therapeutic interventions. A further problem that arises is the effective translatability of the model to human AIH. Many models used disease-unrelated antigens as targets and other models pushed the autoimmune disease in rather unrealistic ways by transferring high numbers of TcR-transgenic T cells. Such circumstances add to the distance of the model to the human disease.

**Figure 1:**
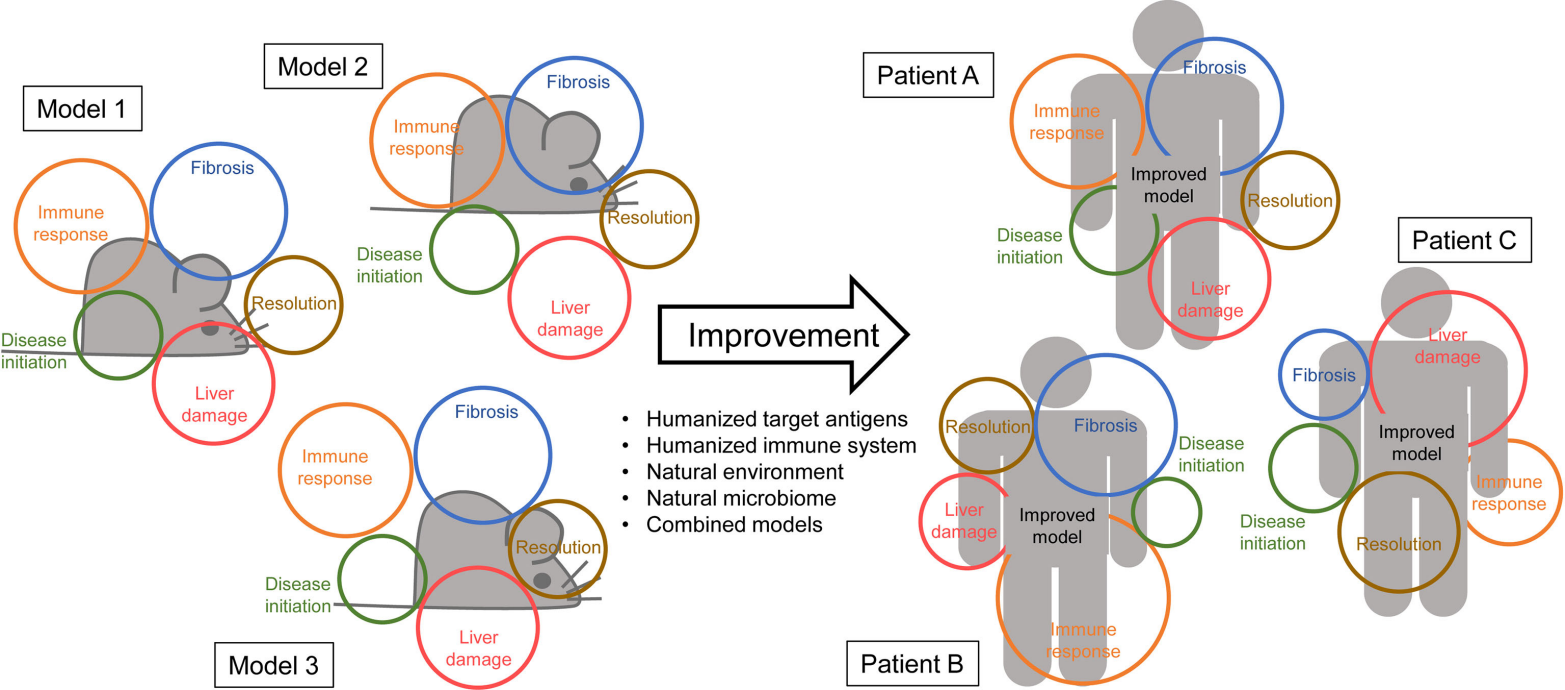
No model is perfect – Left side: Animal models (represented by grey mice) rarely cover all the aspects of the human disease (represented in colored circles). For AIH some models might display a similar immune response as seen in patients but fail in reproducing the actual pathology. Other models might very accurately reflect human fibrosis but use a disease initiation that is unlikely to occur in patients. – Right side: Improvement of the models by combinations, humanization, the use of a natural microbiome and/or environment might increase the area of features covered by the model. Ideally, an improved model might also cover the different disease manifestations in individual patients equally.

We and others have used actual human autoantigens as targets or triggers for experimental AIH (31, 32). The CYP2D6 model developed by us uses wildtype mice of the strains FVB or C57BL/6 that are infected with a recombination deficient adenovirus carrying the gene for human CYP2D6 (Ad-2D6), the major autoantigen in AIH type 2 (31, 33). The wildtype mice express the mouse Cyp homologues, which are similar, but not identical to human CYP2D6. Upon infection with Ad-2D6, that predominantly infects hepatocytes bearing the coxsackie-adenovirus receptor (CAR), the mice display an acute hepatitis that subsequently develops into a chronic AIH-like liver disease (31, 33). As with many models, the CYP2D6 model very accurately reflects some of the characteristics of the human situation, whereas other aspects are only poorly represented. Ad-2D6-infected mice show an interface hepatitis with cellular infiltrates that are dominated by T cells, but also contain neutrophils, B cells, macrophages, and dendritic cells. Interestingly, the CYP2D6-specific immune response is very similar to the one found in patients with AIH type 2. In particular, the mice generate LKM-1-like antibodies that recognize a similar pattern of epitopes as patient antibodies (34). The mice also generate CYP2D6-specific CD4 and CD8 T cells. However, due to the difference in the MHC haplotype between mice and man, the epitope specificity in the mouse is different than in AIH patients (35). The mouse T cell epitopes are located in regions of intermediate homology between CYP2D6 and the mouse Cyp homologues, indicating that the tolerance to the mouse Cyps was likely broken by the similar, but not identical, structures (35). This observation fits well with the concept of molecular mimicry in which a similarity between pathogen and host might result in a breakdown of self-tolerance upon pathogen infection (26). In contrast to the immune response that is rather accurately modelled, the CYP2D6 model only poorly reflects hepatic fibrosis as occurring in patients. Ad-2D6-infected mice display a profound subcapsular fibrosis that is triggered by inflammatory macrophages that arise in the peritoneum upon intraperitoneal infection (36). In contrast, patients with AIH display periportal and bridging fibrosis in close proximity to the cellular infiltrations that are characteristic for an interface hepatitis. Thus, animal models, such as the CYP2D6 model, seem to be only appropriate to study individual aspects of the disease and there seems to be no perfect model for AIH to date (**Figure 1**). In this context it is also important to consider that there are two major forms of AIH, type 1 and type 2. As mentioned above these two types are mainly distinguished on the pattern of autoantibodies patients are generating. Thus, CYP2D6 and FTCD associated models can be considered AIH type 2 models. Since in AIH type 1, autoantibodies, such as ANA, react to several nuclear components, including DNA, centromers, histons, and sn-RNPs, and are also found in other diseases like PBS or systemic sclerosis (SSc) there are no AIH type 1-specific models available. However, since AIH type 1 and type 2 might be just two flavors of the same disease entity (37), focusing on one form only might also contribute to a low translatability. Consequently, a combination of several models might be a solution to the problem. However, the simultaneous use of many different models would require additional resources, take much more time to completion, and add further complexity to the data evaluation.

## Are Therapies at a Standstill?

**Figure 2:**
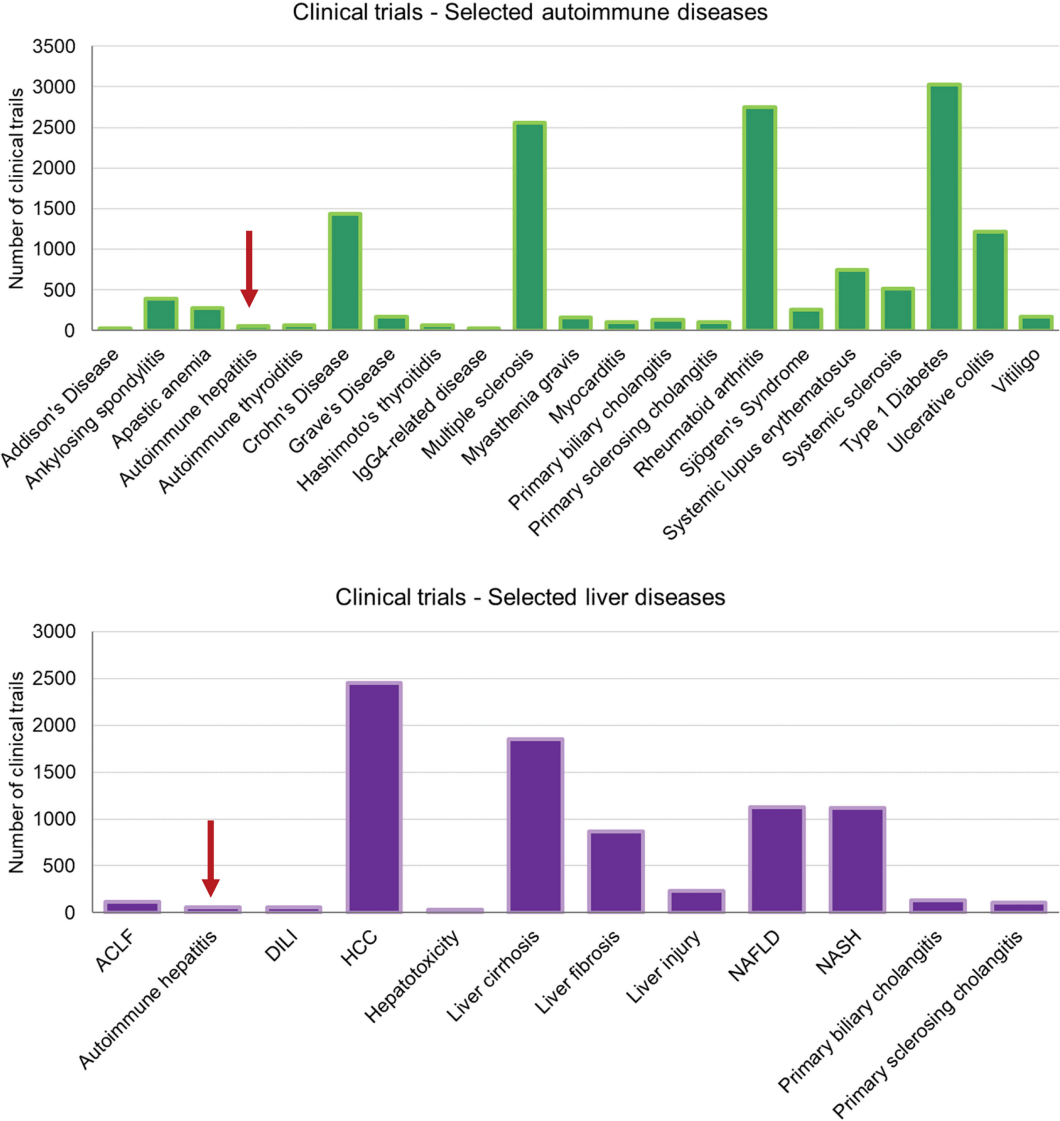
Clinical trials on autoimmune and liver diseases – These charts display the number of clinical trials listed on https://clinicaltrials.gov for selected autoimmune diseases and liver-associated diseases. Note that the total numbers of records listed for “autoimmune disease” and “liver disease” were 10.618 and 10.429, respectively. The search terms were as listed in the axis-label. (ACLF, acute on chronic liver failure; DILI, drug-induced liver injury; HCC, hepatocellular carcinoma; NAFLD, non-alcoholic fatty liver disease; NASH, non-alcoholic steatohepatitis).

All these and many other models not mentioned here have allowed to gain more insight into the pathogenesis of the disease and many critical factors that drive the disease have been identified since. Findings in animal models as well as data obtained from studies in human patients suggest several critical factors driving the disease, including cytokines, such as TNFα, IL-2, IL-6, IL-12, and TGFβ or chemokines, like CXCL9 or CXCL10 (see (38) for more details). However, there are almost no attempts being made to target those factors in patients. It is surprising, or even alarming, to see how few clinical trials on the treatment of AIH are being conducted compared to other autoimmune diseases or other afflictions of the liver (**Figure 2**). One reason for this underrepresentation is of course the low incidence of AIH compared to RA or T1D making it difficult to get a decent cohort of AIH patients for a clinical study. Another cause seems to be a lack of willingness for translation from model to patient. There are two major reasons to this. First, many models have been generated in the context of an immunopathological question but did not involve novel therapeutic interventions. Second, there might be a lack of trust in the traditional models, preventing a continuation to clinical trials. However, there are some exceptions to this rule. In search for alternatives to corticosteroids and cytostatic drugs, mild immunosuppressive drugs, like cyclosporin A (CsA) and tacrolimus, that target IL-2 expression have been investigated in several clinical trials and are now recommended as third-line treatments for AIH when first- and second-line treatments fail (39). Only few patients have been treated with CsA or tacrolimus as a first-line treatment with or without prednisolone (40, 41). Since these studies failed to demonstrate an improvement over standard treatments but revealed some side effects like renal toxicity over time, the application of CsA or tacrolimus might not go beyond a rescue treatment (39).

A few other drugs are under current evaluation in clinical trials or have been used in small off-label studies. Among them are the mammalian target of rapamycin (mTOR) inhibitors sirolimus and everolimus, that target the IL-2-dependent T cell proliferation. However, similarly to CsA, they have been used with limited success only as rescue treatments in some difficult-to-treat patients (39). Interestingly, a multicenter clinical trial (TRANSREG, NCT01988506) is trying the opposite. Instead of blocking IL-2 expression or responsiveness, direct administration of low dose IL-2 is intended to expand Treg in patients with AIH or other autoimmune diseases. Unfortunately, only two AIH patients have been enrolled in this study to date (https://clinicaltrials.gov/ct2/show/NCT01988506). Alternatively, there are attempts to use *in vitro* expanded Treg. A phase I trial intends to isolate Treg (CD4+, CD25+, CD127+) from AIH patients, expand them *in vitro* with anti-CD3/CD28, IL-2 and retinoid acid, and reinject them into the same patients. Unfortunately, there are currently no data available to this study (https://clinicaltrials.gov/ct2/show/NCT02704338). Clearly such studies are aiming at the re-establishment of a healthy immune balance by enhancing the regulatory side, which has been shown to be impaired in patients with AIH (42, 43). In contrast, the immune balance can also be restored by dampening the aggressive side *via* a direct targeting of activated, autoaggressive T cells. Anti-CD3 treatment has been employed in animal models and clinical trials of many autoimmune diseases. Most prominently anti-CD3 antibodies, such as teplizumab have been successfully used to treat early onset T1D (44, 45). Mechanistically, anti-CD3 treatment results in the depletion of some aggressive T cells by inducing activation- induced cell death and the conversion of some T cells to an either unresponsive or regulatory state (46). Another way of pacifying T cells is by inhibiting co-stimulation through blockade of the CD80/86-CD28 interaction with abatacept, a cytotoxic T-lymphocyte-associated protein 4 (CTLA-4)-Ig fusion protein. Abatacept is currently applied as a second-line therapy of RA and is also used to treat other forms of systemic rheumatic diseases (47). Whereas anti-CD3 treatment is currently not considered as therapy for AIH in clinical trials, abatacept is being evaluated in a phase 1 trial in patients with *de novo* or recurrent AIH not responding to standard treatment (https://www.clinicaltrials.gov/ct2/show/NCT04203875).

Surprisingly, besides IL-2, cytokines and other defined inflammatory factors have not been targeted extensively so far. One exception is the neutralization of TNFα that has been successfully conducted in single patients or small cohorts of difficult-to-treat AIH patients using either of the two anti-TNFα antibodies infliximab or adalimumab (48–50). However, several reports have indicated that TNFα neutralization in the context of other autoimmune diseases might result in AIH-like disease (51, 52). The mechanism of how anti-TNFα antibodies might be involved in the pathogenesis of AIH is not yet known. However, it has been suggested that anti-TNFα therapy or a withdrawal thereof might unmask a preexisting condition rather than inducing the disease from scratch (53). As mentioned earlier, IL-12 has not been considered as a target in clinical trials although it has been demonstrated to be a driving factor in experimental AIH (28–30) and a suitable inhibitor (ustekinumab) would be available. Similarly, the chemokines CXCL9 and CXCL10 that rank at the top of the list of pro-inflammatory chemokines (54) and have been demonstrated to orchestrate T cell trafficking to the liver (55) have not yet been targeted. Both CXCR3 ligands are increased in patients with AIH and have been associated with disease severity (56).

Clearly, there is an apparent shortage of clinical trials aiming at a cure for AIH. As suspected before, one reason for this underrepresentation might be a certain lack of trust in preclinical data obtained in animal models. Thus, it is important to improve existing models to get closer to the human disease to cover many aspects of AIH simultaneously and additionally considering factors, such as the microbiome and interindividual differences.

## Improvement: Combination Models

One possibility to cover more features of the human disease would be to combine individual models. Such combinations might even be applied across different (autoimmune) diseases. Hardtke-Wolenski and colleagues used the non-obese diabetic (NOD) mouse that is predominantly used to model spontaneous T1D and induced AIH-like disease by infecting the mice with an Adenovirus encoding either of the two AIH type 2 autoantigens CYP2D6 and FTCD (32). Interestingly, the combination with the immune-regulation deficient NOD mice was a prerequisite for AIH, since only infection of NOD, but not C57BL/6 resulted in AIH-like disease. Another example is the use of a high fat diet to induce a state of non-alcoholic fatty liver disease (NAFLD) in AIH setting. Mice on a high fat diet display typical features of human NAFLD, including hepatic steatosis and typical ballooning. Upon infection with Ad-2D6, just like in the CYP2D6 model, such mice show enhanced AIH-like disease with increased cellular infiltrations and higher frequencies of liver autoantigen-specific T cells (57). Interestingly, the combination of the CYP2D6 model with a high fat diet results in additional AIH-like disease features, such as enhanced peri-portal and bridging fibrosis (57). Yet another combination model was generated by infecting mdr2-/- mice with Ad-2D6. The mdr2-/- mouse is a well-established model for PSC. Such mice carry a deficiency in the ATP binding cassette transporter ABCB4, a phospholipid flippase that causes biliary excretion of phosphatidylcholine, which is required for the packaging of bile acids into micelles. Thus, bile acids produced by mdr2-/- mice leak from the bile ducts to the portal tracts and damage the surrounding cells resulting in a chronic form of biliary liver disease resembling human PSC (58, 59). Upon infection of mdr2-/- mice with Ad-2D6 the mice start showing features of PSC as well as AIH, similar to patients that suffer from a PSC/AIH overlap syndrome (60). The interesting finding towards an improvement of a model for AIH lays in the exacerbation of chronic AIH-like disease and the additional appearance of bridging fibrosis. However, due to the presence of a PSC/AIH overlap those aspects specifically related to PSC, such as bile duct proliferation, might impact the pathogenesis of AIH unpredictably. Thus, it is highly questionable if AIH research would benefit from such a combination model.

At the end, combining two or more models would increase the complexity of the resulting model. However, most human autoimmune diseases are multifactorial and complex as well. The multiple hit theory of autoimmune diseases states that several environmental events might be involved in the etiology of the disease (61). In this context, autoimmune tautology, referring to diseases that share similar immunogenetic mechanisms, has to be considered as well, since an initiation or propagation of similar mechanisms by one or more triggering factors might result in polyautoimmunity/overlap syndromes. Further, interindividual differences for example in the HLA-haplotype, but also in other genes, resulting in a high or low disease susceptibility add to the complexity.

## Improvement: Humanized Models

The generation of humanized models has been used to mimic many diseases. In AIH the use of human liver autoantigens, such as CYP2D6 or FTCD, rather than human disease unrelated antigens can be considered as a shy attempt to humanize an AIH model. However, most of the currently used humanized models for autoimmune diseases go many steps further in trying to recreate the human immune system or at least parts of it. Such models either use immunodeficient mice that are repopulated with human immune cells, transgenic MHC humanized mice, or combinations thereof (62, 63). In immunodeficient mouse lines, such as the well-known severe combined immunodeficient (SCID) mouse or the lymphopenic IL-2 receptor common gamma chain (IL-2rγ) knock-out mouse, every individual mouse requires an engraftment by hematopoietic stem cells or entire organs, such as thymus or fetal liver, to establish parts of a human immune system (63). This circumstance is somewhat aggravating especially if large numbers of mice are to be studied. However, such humanized models offer the big advantage that almost all lineages of human immune cells can be investigated in the mouse.

In a much simpler approach, MHC humanized mice usually just express those human leukocyte antigen (HLA) class I or II alleles that are considered predominant risk factors for the individual disease, such as HLA-DR3, -DR4, and -DR7 in AIH. To avoid interference with endogenous mouse MHC molecules, such mice are often generated on a corresponding MHC class I or II deficient background. Thus, such mice allow to recreate an antigen presentation similar to the one observed in human patients. Yuksel et al. generated HLA-DR3 humanized mice on a NOD background and found that the presence of the human risk allele indeed exacerbated AIH-like disease after DNA-vaccination with a plasmid encoding CYP2D6 and FTCD (64). Compared to normal NOD mice, HLA-DR3-positive mice showed on the one hand higher serum aminotransferase levels, cellular infiltrations, and fibrosis scores and on the other hand enhanced antibody titers, a higher T cell activity, and a lower frequency of Treg (64). Very similar findings were reported by the same authors for CYP2D6/FTCD DNA-vaccinated HLA-DR4 humanized NOD mice (65). Thus, the reported data indicate that the presence of human HLA risk allels indeed changed the immune response as well as the overall pathology in the mouse.

Surely, simple HLA-humanized mice do not recreate the human immune system in the same extent as it is present in repopulated immunodeficient mice. However, since gene rearrangement processes shape an antibody and TcR repertoire of almost infinite numbers of different antigen-specificities in mouse and men, the repertoire adapts to the present MHC haplotype. Thus, the presence of human HLA molecules also results in an antibody and TcR repertoire that is different from the one present in wildtype mice. In recent studies, humanized models using repopulated immunodeficient mice have been combined with HLA-humanized mice, which obviously brings the model even closer to the human situation (63). However, such a combination also adds to the complexity of the model and the experimental effort. In addition, analyzing immunocompromised mice, even with a reconstituted human immune system, requires working in a protected environment that might reverse the achieved proximity to the human situation.

## Improvement: Natural Microbiome

An interesting aspect of the abovementioned work by Yuksel et al. is that HLA-DR3 humanized NOD mice displayed a composition of the gut microbiota that was strikingly different from that of wildtype NOD mice (64). From that study it was not clear whether the mere presence of the HLA-DR3 is shaping the microbiota differently or whether the exacerbation of the AIH-like disease has an influence on the microbiome composition. However, the study brings another variable into the equation, the microbiome. Over the last decades evidence has grown that the microbiome plays an important role in the development of autoimmune diseases (66). Due to its anatomic location the liver receives about 70% of its blood supply *via* the portal vein and stands in close contact with the gastrointestinal tract through the enterohepatic pathway. Thus, besides nutrients and xenobiotics, gut microbiota and their metabolites are in constant exchange between liver and gut. The proximity between gut and liver may also be the reason why there is such a high proportion of patients with PSC who also suffer from inflammatory bowel disease (IBD) (67). Interestingly, dysbiosis, a disruption of the microbiota homeostasis, has been associated with many autoimmune diseases (68). Thus, it comes as no surprise that dysbiosis has been shown to affect ALD as well as other liver diseases, such as NAFLD (68, 69). In a very recent study, Liang et al. were able to attenuate AIH progression in an experimental AIH model with mice harboring dysbiosis by transplantation of fecal microbiota from normal C57BL/6 mice (70). In contrast translocation of the gut pathobiont, Enterococcus gallinarum, to the liver and other organs of autoimmune-prone hosts drives the autoimmune pathogenesis (71). Thus, since there is such a strong link between gut and liver, the question arises whether artificial laboratory conditions as present in most animal facilities influence the outcome of experiments conducted in mouse models. Such discrepancy might as well influence the translatability of mouse data to the human situation. In this context a recent study by Rosshard and colleagues brought some interesting new insight. Laboratory mice carry a rather peculiar microbiome that is dependent on the breeding and experimental facilities as well as the mouse strains. Compared to wild mice the microbiome of laboratory mice lacks diversity, which might account for a biased immune response. To overcome such a bias Rosshart et al. have recolonized the microbiome of laboratory mice with the gut microbiome of wild mice that were captured in horse stables located in the region of their lab at the NIH in Bethesda, MD (72). Such reconstituted laboratory mice with natural microbiota exhibited an increased overall fitness compared to regular laboratory mice. This enhanced fitness was evidenced by an increased survival rate following infection with influenza virus as well as an increased resistance against mutagen/inflammation-induced colorectal tumorigenesis (72). In a follow-up study the authors went one step further and generated so-called wildling mice that contain the microbiome of wild mice. They have achieved this by transferring embryos of laboratory mice into pseudo-pregnant wild mice carrying a natural microbiome (73). They then used these mice to investigate if a bias microbiome influences the translatability of mouse data to the human situation. In particular, they investigated the use of the superagonist anti-CD28 antibody teralizumab (TGN1412) and an anti-TNFα antibody. Both Treg activation *via* CD28 and TNFα blockade failed in clinical studies in healthy volunteers and patients with severe sepsis, respectively. Anti-CD28 therapy was successful in activating Treg in normal laboratory mice, but resulted in a strong cytokine activation (cytokine storm) and a lack of Treg expansion in wildling mice (73), just as in the failed clinical study by TeGenero (74, 75). Similarly, TNFα blockade was effective in preventing lethal endotoxemia in laboratory mice (76), but the administration of the TNFR75-IgG fusion protein etanercept even increased the mortality of patients with septic shock (77). In contrast to laboratory mice, wildling mice treated with an anti-TNFα antibody displayed a lower survival rate in a lethal endotoxemia setting than wildling mice administered with an isotype-matched control antibody (73). These data clearly demonstrate that the microbiome plays an important role in the outcome of a therapeutic intervention in animal models and that dysbiosis as present in most laboratory mice might be one factor that prevents a successful translation into patients.

## Improvement: Natural Environment

A natural microbiome without dysbiosis is only one out of many factors that is aberrant in an artificial laboratory setting. Factors, such as room temperature, light-dark cycle, humidity, sterile or semi-sterile housing conditions (hygiene), and food composition are often neglected in preclinical studies. It is well known that the hygiene in the animal facility has an impact on the incidence of autoimmune diseases. For example, the incidence of T1D in NOD mice is dependent on the hygiene, with mice showing a higher T1D incidence in a clean environment (78). Simple factors, such as the room temperature, are often suited for the comfort of a fully dressed investigator rather than the experimental animal. The thermoneutral zone for most mouse strains during the day is 30–32°C. Thus, mice often suffer from cold stress that is characterized by an increased heart rate and an elevated basal metabolic rate (79). Interestingly, the adaptation to cold stress also involves the production of higher levels of endogenous glucocorticoids which might influence the outcome of immunological studies (79). Similarly, the light-dark cycle is mostly set to 12 hours of light and 12 hours of darkness and is not changed seasonally. It mainly the researchers performing the experiments at daytime when they are mainly active. However, mice are naturally more active at night and therefore disturbed during their resting period through experimental handling at daytime. Further, nutrition is a factor that should be considered even though most animal facility nowadays use similar standard chow. A whole field of “nutri-epigenetics” is focusing on the effect of maternal diet and early childhood nutrition in the context of autoimmunity (80). Large clinical studies, such as “the environmental determinants of diabetes in the young (TEDDY) study intend to identify environmental factors, like infectious agents, nutrition, or psychosocial factors that might trigger T1D in susceptible individuals (https://clinicaltrials.gov/ct2/show/NCT00279318) (81). Unfortunately, a similar large-scale study has not been performed for AIH.

Another factor that is profoundly different in most laboratory mice and humans is the genetic diversity. Most rodent models use inbred strains of mice, rats, guinea pigs, or rabbits. On the one hand the use of an inbred population with an almost identical defined genome has the advantage of low interindividual diversity, which results in higher rate of significance and reproducibility. On the other hand, an inbred population is the exact opposite of the situation found in human individuals, who, with the exception of “identical” twins, are closer to represent two different mouse strains rather than two individuals of the same strain. Therefore, many models use outbred strains that indeed behave differently from inbred strains. Studies on allograft rejection, intestinal inflammations (inflammatory bowel disease (IBD), Crohn’s disease), or neuroinflammation (EAE), all resulted in different outcomes in inbred and outbred strains (82).

In this context one also should ask the question whether the predominant laboratory animal, the mouse, is indeed an adequate organism to study human AIH. Traditionally, mice have been used to study autoimmune diseases, since the immune system of the mouse shares many similarities with the human immune system and still breeding and keeping of mice as well as the generation of transgenic variants is much easier than for dogs, pigs, macaques, or non-human primates. In contrast, toxicology studies are often conducted in rats, rabbits, or dogs and studies of infectious diseases, such as AIDS, are still often performed in non-human primates. However, the use of non-human primates, in a large scale for basic or translational research is from an ethical as well as financial point of view not an option. Nevertheless, translation of mouse models might be more successful when performed in two steps including for example small numbers of macaques as intermediate.

## Summary and Outlook

It seems evident that the use of current animal models for AIH helped to get a better insight into the pathogenesis of the disease rather than provided a basis for therapeutic intervention. On the one hand the models have not yet been extensively used to evaluate therapeutic interventions and on the other hand the general low success rate of translatability of pre-clinical data to patients in other diseases prevented the final step into the clinic. However, one has also to consider that for low-prevalence diseases, like AIH, the hurdles to get into clinical trials, such as recruitment of patients, financing, and finding of clinical partners, seem to be much higher as for other autoimmune diseases. Thus, it is important that there is growing trust in pre-clinical data from animal models. Lack of translatability or a low success rate is often used by animal right activists in political discussions about a possible complete ban of research on animals. Very recently, on Feb. 14th, 2022, the people of Switzerland voted against a ban of research on animals and human individuals by a rather high margin (https://www.bk.admin.ch/ch/d/pore/va/20220213/can651.html). Indeed, the political discussions beforehand centered on the translatability of data from intervention studies in animal models to clinical studies in patients. Unfortunately, such discussions are mostly dominated by incomplete data on the topic of translation from both sides. A recent study systematically assessed concordance rate between animal and human studies in attempt to answer how the overall animal-to-human success rate really is (83). The meta-study included reviews and all other types of “umbrella”-studies of meta-data quantitatively comparing the translational results of studies including at least two species with one being human. Unfortunately, the final outcome of this study was inconclusive, and the authors stated that translational success is currently unpredictable.

Lack of translatability results in frustration of both hopeful patients as well as scientists and clinicians involved. Nevertheless, an effort has to be made to understand the reasons why the translation failed. Therefore, reverse translation from the clinical study back to the animal model might help to improve the model and/or gain additional knowledge about the mechanisms of disease. Here, we want to come back to the aforementioned failed phase I trial on Treg activation with the CD28 superagonist teralizumab (TGN1412) by TeGenero (74, 75). Follow-up studies have revealed additional factors that need to be considered in these models. For example, in contrast to human individuals, most laboratory animals live in a rather sterile environment and thus carry much lower frequencies of memory T and B-cells. Thus, whereas in rodents teralizumab administration resulted in an expansion of Treg, in humans the activation of dormant memory T cells and their unregulated migration into extra-lymphoid tissue (84) resulted in the observed cytokine storm with all its detrimental consequences. In addition, it has been found that although teralizumab showed a similar affinity for mouse, monkey, and human CD28, the induced Ca^2+^ flux was much lower in monkey T cells resulting in a decreased activation compared to human T cells (85). Reversed translation also helped to find novel targets in MS therapy. Ustekinumab was successful in preventing experimental autoimmune encephalomyelitis (EAE) in mice and marmosets, but failed in clinical studies with MS patients (86). Subsequent studies aiming at finding reasons for the failure revealed CD3+ CD4/5+ CD56+ cytotoxic T as possible new targets since these cells were the main drivers of EAE progression and antibody-independent demyelination (87). On a larger scale of reverse translation, several failures in clinical studies targeting T cells resulted in a re-evaluation of the pathogenic role of immune cell subsets in MS. B cells that for a long time have been considered as mere antibody producers have now moved into the center of attention. B cell depletion through administration of anti-CD20 antibodies, such as rituximab or ofatumumab, or blockade of B cell differentiation by antibodies to B lymphocyte stimulator (BlyS), also known as B cell activating factor (BAFF), showed a remarkable efficacy both in animal models as well as in clinical trials (88). It is to hope that more of such reverse translations will also be applied on AIH in the future.

**Figure 3:**
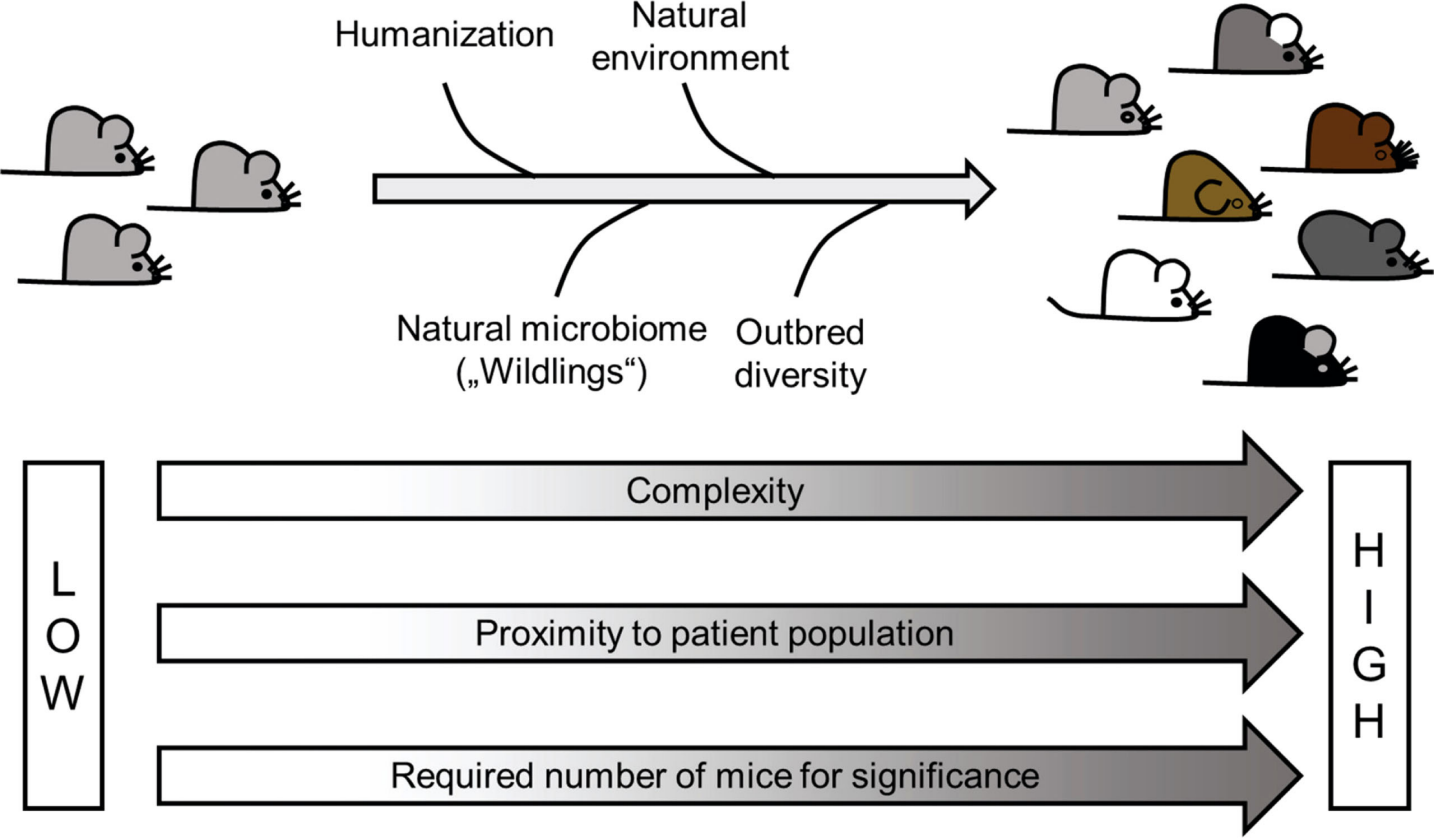
Improvement of animal models – Most of the current AIH models use traditional inbred mouse strains housed in a sterile and artificial environment at surrounding conditions that often fit fully dressed scientists rather than the experimental animals. This obvious contrast to the life of patients might be one of the reasons for a low success rate in translation. Improvement such as humanization, use of outbred strains, as well as a more natural environment and an absence of dysbiosis might bring the models closer to the situation in patients. However, such improvement would also come with a price tag, namely a higher complexity and higher number of animals required to achieve significance.

Even prior to reverse translation, improvement of current models can be achieved by multiple modifications, including further humanization of the models, consideration of the microbiome, attention to a more natural housing, and introduction of an outbred mouse colony (**Figure 3**). Thus, one can ask the question whether we should now all work with multi-humanized wildling mice derived from outbred strains that have been challenged with several environmental factors, including pathogens and xenobiotics, and are kept under natural outdoor conditions. Possibly that would not be such a very well-reasoned idea. However, it might already be a good start to keep all the factors that might influence the outcome of animal experiments in mind and in addition at least increase the temperature in the animal facility just by a few degrees. Even more important would be to use current models to evaluate explicitly therapeutic interventions and then have the courage to translate the obtained results to the clinic.

## Author Contributions

UC and EH wrote the review article and designed the figures. All authors contributed to the article and approved the submitted version.

## Conflict of Interest

The authors declare that the research was conducted in the absence of any commercial or financial relationships that could be construed as a potential conflict of interest.

## Publisher’s Note

All claims expressed in this article are solely those of the authors and do not necessarily represent those of their affiliated organizations, or those of the publisher, the editors and the reviewers. Any product that may be evaluated in this article, or claim that may be made by its manufacturer, is not guaranteed or endorsed by the publisher.

## Abbreviations

ALD, autoimmune liver diseases; AIH, autoimmune hepatitis; PBC, primary biliary cholangitis; PSC, primary sclerosing cholangitis; IAC, immunoglobulin G4 (IgG4)-associated cholangitis; ANA, anti-nuclear antibodies; SMA, anti-smooth muscle autoantibodies; LKM-1, liver-kidney microsomal antibodies type 1; AMA, anti-mitochondrial antibodies; EAH, experimental autoimmune hepatitis; DILI, drug-induced liver injury; HSC, hepatic stellate cells; LSEC, liver sinusoidal endothelial cells; MBP, myelin basic protein; MS, multiple sclerosis; HBsAg, hepatitis B virus surface antigen; Alb, albumin; CTL, cytotoxic T-lymphocytes; TcR, T cell receptors ().; LCMV, lymphocytic choriomeningitis virus; GP, glycoprotein; NP, nucleoprotein; T1D, type 1 diabetes; TTR, transthyretin; FTCD, formiminotransferase cyclodeaminase; CYP2D6, cytochrome P450 2D6; AAV, adeno-associated viral vector; Ad-2D6, adenovirus carrying the gene for human CYP2D6; CsA, cyclosporin A; CTLA-4, cytotoxic T-lymphocyte-associated protein 4; NOD, non-obese diabetic; NAFLD, non-alcoholic fatty liver disease; ABCB4, ATP binding cassette transporter 4; SCID, severe combined immunodeficient; IL-2rγ, IL-2 receptor common gamma chain; HLA, human leukocyte antigen; IBD, inflammatory bowel disease; TEDDY, the environmental determinants of diabetes in the young; EAE, experimental autoimmune encephalomyelitis; BlyS, B lymphocyte stimulator; BAFF, B cell activating factor.

## Glossary

**Table:**

ALD	autoimmune liver diseases
AIH	autoimmune hepatitis
PBC	primary biliary cholangitis
PSC	primary sclerosing cholangitis
IAC	immunoglobulin G4 (IgG4)-associated cholangitis
ANA	anti-nuclear antibodies
SMA	anti-smooth muscle autoantibodies
LKM-1	liver-kidney microsomal antibodies type 1
AMA	anti-mitochondrial antibodies
EAH	experimental autoimmune hepatitis
DILI	drug-induced liver injury
HSC	hepatic stellate cells
LSEC	liver sinusoidal endothelial cells
MBP	myelin basic protein
MS	multiple sclerosis
HBsAg	hepatitis B virus surface antigen
Alb	albumin
CTL	cytotoxic T-lymphocytes
TcR	T cell receptors ().
LCMV	lymphocytic choriomeningitis virus
GP	glycoprotein
NP	nucleoprotein
T1D	type 1 diabetes
TTR	transthyretin
FTCD	formiminotransferase cyclodeaminase
CYP2D6	cytochrome P450 2D6
AAV	adeno-associated viral vector
Ad-2D6	adenovirus carrying the gene for human, CYP2D6
CsA	cyclosporin A
CTLA-4	cytotoxic T-lymphocyte-associated protein 4
NOD	non-obese diabetic
NAFLD	non-alcoholic fatty liver disease
ABCB4	ATP binding cassette transporter 4
SCID	severe combined immunodeficient
IL-2r&gamma	IL-2 receptor common &gamma chain
HLA	human leukocyte antigen
IBD	inflammatory bowel disease
TEDDY	the environmental determinants of diabetes in the young
EAE	experimental autoimmune encephalomyelitis
BlyS	B lymphocyte stimulator
BAFF	B cell activating factor
